# SARS-CoV-2 Infection Alters the Immune Microenvironment in Lung Cancer Patients Undergoing Immunotherapy and Affects Treatment Outcomes

**DOI:** 10.3390/v17101314

**Published:** 2025-09-28

**Authors:** Yanjing Peng, Panjian Wei, Meng Gu, Guirong Wang, Teng Ma, Jinjing Tan

**Affiliations:** 1 Cancer Research Center, Beijing Chest Hospital, Capital Medical University, Beijing 100069, China; pengyj77@163.com (Y.P.); gmaixy1314@126.com (M.G.); mateng82913@163.com (T.M.); 2 Department of Laboratory Medicine, Beijing Chest Hospital, Capital Medical University, Beijing 100069, China; 69547223@sohu.com (P.W.); wangguirong1230@ccmu.edu.cn (G.W.)

**Keywords:** COVID-19 and lung cancer, Immune Checkpoint Inhibitors (ICI), cytokine dynamics, Progression-Free Survival (PFS), immune landscape alteration

## Abstract

Background: The COVID-19 pandemic prompted investigation into the interaction between SARS-CoV-2 infection and immune checkpoint inhibitor (ICI) therapy in lung cancer patients. Understanding this interplay is crucial for optimizing cancer immunotherapy. Methods: A retrospective analysis was conducted on lung cancer patients, characterizing changes in peripheral immune cells and plasma cytokines (including IL-10 and IL-12p70) before, during, and after SARS-CoV-2 infection. Progression-free survival (PFS) was compared between ICI-treated patients with and without COVID-19. Cytokine dynamics were further analyzed in a non-infected cohort. Results: SARS-CoV-2 infection induced a prolonged systemic cytokine storm, with elevated IL-10 and IL-12p70 levels and reduced monocyte proportions lasting up to 10 weeks post-recovery. Despite this immune perturbation, COVID-19 did not impair long-term PFS; instead, a transient improvement in disease control was observed in infected patients. In non-infected patients, sustained or increased IL-10 and IL-12p70 levels during ICI therapy were associated with longer PFS (*p* < 0.05). Conclusions: SARS-CoV-2 infection transiently alters the immune landscape in lung cancer patients without compromising ICI efficacy. The sustained elevation of IL-10 and IL-12p70 may contribute to short-term clinical benefits. Monitoring cytokine dynamics could serve as a prognostic tool for predicting ICI response.

## 1. Introduction

Just as immunotherapy began to revolutionize the lung cancer treatment landscape by harnessing the power of the body’s own immune system [1], the COVID-19 pandemic introduced a formidable new variable [2,3]. For millions of already vulnerable lung cancer patients, viral infection transformed from a common risk into a potential catalyst that could alter the entire course of their anti-cancer therapy. This new reality posed a critical question to oncologists and researchers worldwide: does the acute inflammatory storm ignited by SARS-CoV-2 ultimately help or hinder the delicate immunological battle waged by immunotherapy against lung cancer [3,4]?

The interaction between immunotherapy, particularly immune checkpoint inhibitors (ICIs), and COVID-19 is fraught with uncertainty, presenting a complex dichotomy. On one hand, viral infection could theoretically act as an immune adjuvant [3,5]. There have been several documented cases of spontaneous tumor regression in cancer patients following SARS-CoV-2 infection, suggesting that the acute inflammatory response may reactivate a dormant anti-tumor immunity by altering the tumor microenvironment’s (TME) cellular and cytokine profile [3,6].

On the other hand, a more compelling body of evidence points toward potential antagonism and heightened risk [7]. The fundamental principle of ICIs is to block inhibitory pathways like PD-1/PD-L1, thereby releasing the brakes on T cells to attack tumors, which are often characterized as sites of chronic inflammation [8]. SARS-CoV-2 infection, however, induces an acute, systemic hyperinflammatory state [9]. This raises concerns that the two processes could dangerously synergize. The pathology of severe COVID-19 involves immune dysregulation, including lymphopenia and the so-called “cytokine storm,” which bears a striking resemblance to the mechanisms underlying immune-related adverse events (irAEs) [10,11,12].

Indeed, studies have suggested that patients with pre-existing irAEs have a significantly higher risk of developing COVID-19 pneumonia [13]. Furthermore, a major meta-analysis found that while prior ICI use did not increase the risk of SARS-CoV-2 infection or mortality, it was significantly associated with an increased likelihood of developing severe or critical disease [14,15]. The diagnostic challenge is also significant, as COVID-19 pneumonitis and ICI-induced pneumonitis can present with similar clinical and radiological features but require vastly different treatment strategies [16]. In rare but severe instances, COVID-19 has even been reported to trigger life-threatening irAE-cytokine release syndrome (CRS) [17,18]. Conversely, several retrospective analyses demonstrate that ICI exposure does not increase the incidence of severe COVID-19 events in lung-cancer patients and therefore should not prompt deferral or discontinuation [19].

Despite these risks, the clinical picture remains contested, with some studies reporting that ICI treatment does not negatively impact survival outcomes in cancer patients with COVID-19. Weimin Li reported three cases, in which patients with metastatic non-small-cell lung cancer who contracted SARS-CoV-2 at the outset of ICI therapy continued treatment without interruption, showing only mild COVID-19 symptoms and no apparent loss of therapeutic benefit [20]. Similarly, data from the VATICO trial [21] and a case series by Xenia Elena Bacinschi [22] suggest that immune-checkpoint inhibition may even confer a clinical advantage in COVID-19 patients, although confirmatory studies are still required. A comprehensive review by Steven C Ka [23] acknowledges the pivotal role of immunotherapy in metastatic lung cancer while underscoring the new risks imposed by the pandemic; nevertheless, it leaves open the question of whether SARS-CoV-2 ultimately compromises the long-term efficacy of ICIs.

Much of the research during the pandemic’s peak understandably focused on acute management protocols and short-term risks [24]. As the global health crisis has subsided, a crucial window has opened to investigate the long-term consequences of this viral encounter on immunotherapy efficacy, offering a new perspective on this complex interaction [25]. In this study, we aimed to address these lingering questions. We first dissected the profound and dynamic changes that SARS-CoV-2 induced within the immune systems of lung cancer patients. By analyzing whole blood and plasma samples collected before, during, and after infection, we mapped the impact of the virus on immune cell populations and cytokine levels, providing a detailed picture of the altered tumor immune microenvironment (TIME). Subsequently, we connected these immunological shifts to the ultimate clinical outcome: the long-term efficacy of ICIs. We compared the progression-free survival (PFS) of ICI-treated lung cancer patients who contracted COVID-19 with a control group of uninfected patients. Finally, we explored the relationship between plasma cytokine fluctuations and PFS in all patients, assessing the potential of using cytokine dynamics as a predictive biomarker for monitoring immunotherapy response. This work aimed to shed new light on the intricate triad of infection, immunity, and cancer, with broader implications for cancer management in the context of concurrent infectious diseases.

## 2. Materials and Methods

### 2.1. Study Objects

For the analysis of immune system dynamics, this study enrolled 30 patients who had previously received treatment at the Department of Oncology, Beijing Chest Hospital, Capital Medical University, and were re-hospitalized due to their first SARS-CoV-2 infection between December 2022 and May 2023. The study was approved by the Ethics Committee of Beijing Chest Hospital, Capital Medical University (Approval No. LW-2025-023). Nasopharyngeal swabs were collected from all patients upon admission for molecular detection of SARS-CoV-2 RNA (RealLine SARS-CoV-2 kit, Bioron Diagnostics GmbH, Römerberg, Germany). Inclusion criteria were as follows: ① diagnosis of COVID-19 according to the “Diagnosis and Treatment Protocol for Novel Coronavirus Pneumonia (Trial Version 9)”; ② diagnosis of lung cancer conforming to “The 2021 WHO Classification of Thoracic Tumors”; ③ availability of whole blood immunophenotyping or plasma cytokine analysis results; ④ complete clinical data before and after immunotherapy; ⑤ complete clinical information; and ⑥ absence of other major underlying conditions (e.g., severe cardiac disease, autoimmune disorders, uncontrolled infections). Exclusion criteria were: ① concurrent bacterial or other viral infections; ② pre-existing immune system diseases; and ③ patients not experiencing their first SARS-CoV-2 infection. We collected clinical data from whole blood immunophenotyping and plasma cytokine analysis during the patients’ hospitalization for COVID-19, defined as within 7 days of infection onset (intra-infection). We also retrieved their test data from within 10 weeks before infection (pre-infection) and within 10 weeks after infection (post-infection).

To analyze the survival outcomes of ICI-treated patients, we screened individuals who received inpatient treatment at the Department of Oncology, Beijing Chest Hospital, Capital Medical University, between December 2022 and May 2023. The specific inclusion and exclusion criteria were: ① diagnosis of lung cancer according to “The 2021 WHO Classification of Thoracic Tumors,” with or without a first-time SARS-CoV-2 infection; ② for patients with COVID-19, diagnosis conformed to the “Diagnosis and Treatment Protocol for Novel Coronavirus Pneumonia (Trial Version 9)”; ③ patients were treated with ICIs, and those with COVID-19 must have contracted their first infection during the course of ICI therapy; ④ patients receiving ICIs as neoadjuvant therapy were excluded; ⑤ patients with non-pulmonary primary tumors were excluded, though those with distant metastases from primary lung cancer were included; ⑥ patients with co-existing immune system diseases were excluded. A total of 94 eligible patients were selected from an initial pool of 404. These patients were divided into two groups based on their COVID-19 status: the lung cancer with COVID-19 group and the lung cancer-only group. A survival analysis of progression-free survival (PFS) was conducted for both groups, with the observation period starting from the first administration of ICI therapy and ending at drug resistance or disease progression.

### 2.2. Whole Blood Immunophenotyping

Sample processing was performed using the BD Multitest™ 6-Color TBNK (BD, Franklin Lakes, NJ, USA) reagent kit according to the manufacturer’s instructions. Briefly, 50 µL of fresh whole blood was added to flow cytometry tubes containing pre-aliquoted antibody cocktails. The tubes were gently vortexed and incubated at room temperature in the dark for 15 min. Following incubation, 450 µL of freshly prepared 1X BD FACSTM Lysing Solution (BD, Franklin Lakes, NJ, USA) was added, and the mixture was gently vortexed and incubated for another 15 min at room temperature in the dark before analysis. Additional fluorescently labeled antibodies against HLA-DR, CD25, CD127, and CD14 were purchased from BD Biosciences (BD, Franklin Lakes, NJ, USA). The following immune cell subsets were quantified as relative percentages: total lymphocytes (CD45+), total T cells (CD3+/CD45+), helper T cells (Th; CD3+CD4+/CD45+), regulatory T cells (Tregs; CD4+CD25+CD127low/CD4+), cytotoxic T cells (CTLs; CD3+CD8+/CD45+), natural killer (NK) cells (CD3-CD16+56+/CD45+), NKT cells (CD3+CD56+/CD45+), total B cells (CD3-CD19+/CD45+), HLA-DR+ classic monocytes (CD14+HLA-DR+/CD3−CD19−), and activated T cells (CD3+HLA-DR+/CD3+). The CD4+/CD8+ ratio was also calculated. All samples were acquired and analyzed on a BD LSRFortessa™ flow cytometer (BD, Franklin Lakes, NJ, USA) using BD FACSDiva™ Software v8.0.2. The gating strategy is detailed in Supplementary Figure S1.

### 2.3. Plasma Cytokine Analysis

The absolute concentrations of IL-1β, IL-2, IL-4, IL-5, IL-6, IL-8, IL-10, IL-12p70, IL-17A, IL-17F, IL-22, TNF-α, TNF-β, and IFN-γ in patient plasma were measured using a cytokine detection kit (Tianjin King-Biosino Biotechnology Co., Ltd., Tianjin, China) based on an immunofluorescence luminescence assay, following the manufacturer’s protocol. In brief, 20 µL of serially diluted calibrators or plasma samples were added to centrifuge tubes containing a capture microsphere mixture and mixed thoroughly. A detection antibody mixture was then added, and the tubes were incubated on a shaker for 2 h at room temperature, protected from light. Subsequently, streptavidin-phycoerythrin (SA-PE) was added, followed by a 30 min incubation on a shaker in the dark. After two washes with buffer solution, the microspheres were resuspended in 200 µL of buffer for analysis. Samples were acquired on a BD FACSCanto™ II system using BD FACSDiva™ Software v8.0.2. Data were analyzed using FCAP Array™ Software v3.0.19.2091.

The reference ranges for the cytokines were as follows: IL-1β (0–3.40 pg/mL), IL-2 (0–6.64 pg/mL), IL-4 (0–4.19 pg/mL), IL-5 (0–4.15 pg/mL), IL-6 (0–11.09 pg/mL), IL-8 (0–15.71 pg/mL), IL-10 (0–4.5 pg/mL), IL-12p70 (0–10.18 pg/mL), IL-17A (0–4.74 pg/mL), IL-17F (0–4.66 pg/mL), IL-22 (0–3.64 pg/mL), TNF-α (0–4.50 pg/mL), TNF-β (0–2.54 pg/mL), and IFN-γ (0–4.43 pg/mL).

### 2.4. Statistical Analysis

Data were presented as counts and percentages for categorical variables, mean and standard deviation (SD) for parametric variables, and median and interquartile range (IQR) for non-parametric variables. Categorical variables were compared using the chi-square test. Student’s *t*-test, Mann–Whitney U test, or paired *t*-test were used to compare changes in cytokine levels and immune cell subset proportions across different SARS-CoV-2 infection stages. Kaplan–Meier survival curves were plotted, and survival differences were assessed using the log-rank and Gehan-Breslow-Wilcoxon tests. For cytokine level analysis, patients were stratified into a “cytokine-increased” group and a “cytokine-not-increased” group using a cutoff of a >10% increase from pre-treatment levels. Hazard ratios (HRs) and 95% confidence intervals (CIs) were calculated using the Cox proportional hazards model. Cytokines with a *p*-value < 0.1 in univariate analysis were included in the multivariate model. For all analyses, a two-sided *p*-value < 0.05 was considered statistically significant. All statistical analyses were performed using GraphPad software (version 26.0.0), and graphs were generated with GraphPad Prism (version 9.0.0).

## 3. Results

### 3.1. The Immune System Under Siege: Mapping the Cytokine and Cellular Shockwave of COVID-19

**Figure 1:**
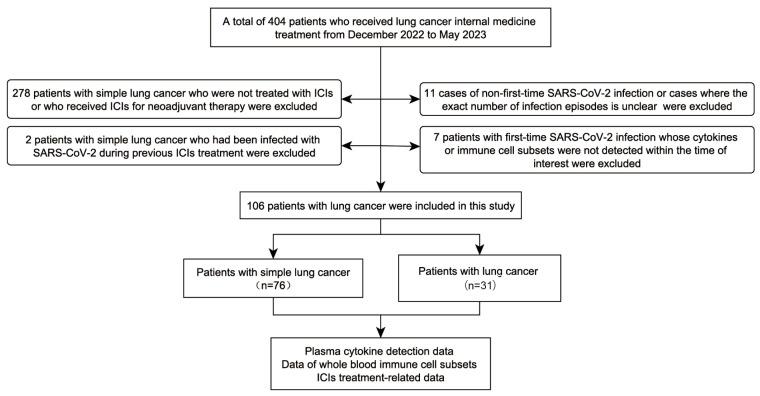
Flowchart of study population selection.

This study aimed to investigate the impact of COVID-19 on patients undergoing immunotherapy. We selected eligible participants from a cohort of patients hospitalized during a local COVID-19 surge between December 2022 and May 2023. Based on predefined inclusion and exclusion criteria, a total of 31 patients with lung cancer and concurrent COVID-19 were enrolled in this study (Figure 1). Among the 31 enrolled patients, 22 were male and 7 were female; the mean age was 64.1 ± 7.9 years.

To analyze the changes in peripheral blood immune cell subsets and cytokines in lung cancer patients before, during, and after SARS-CoV-2 infection, we collected their respective test results at these time points. The “pre-infection” period was defined as 3 to 10 weeks before the confirmed SARS-CoV-2 diagnosis. The “during-infection” period was defined as within 7 days after the confirmed diagnosis, and the “post-recovery” period was defined as 4 to 10 weeks after recovery from the infection. Among the enrolled patients, 17 had their peripheral blood immune cell subsets measured at different time points, with 6 of these patients having data available for all three time points. The demographic and clinical information for these patients is detailed in Supplementary Table S1. Concurrently, a total of 31 patients with lung cancer and COVID-19 had plasma cytokine levels measured at various time points. Among them, 8 patients had complete cytokine data across all three periods (pre-infection, during-infection, and post-recovery). The demographic and clinical characteristics of the patients who underwent plasma cytokine analysis are summarized in Supplementary Table S2.

### 3.2. The Initial Cytokine Storm

**Figure 2:**
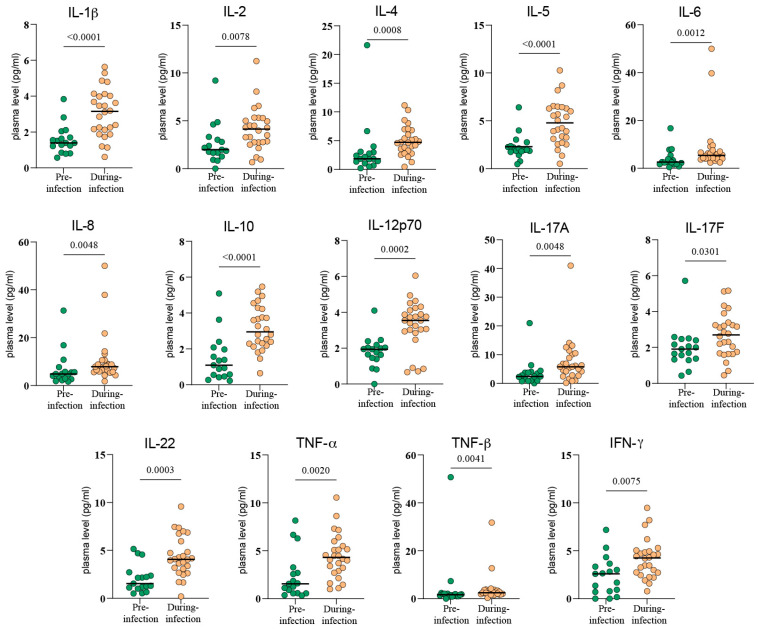
Comparison of plasma cytokine levels in lung cancer patients pre-infection (*n* = 17; green) and during infection (*n* = 26; yellow) of SARS-CoV-2, showing a widespread and significant increase. Black bars indicate mean values.

**Figure 3:**
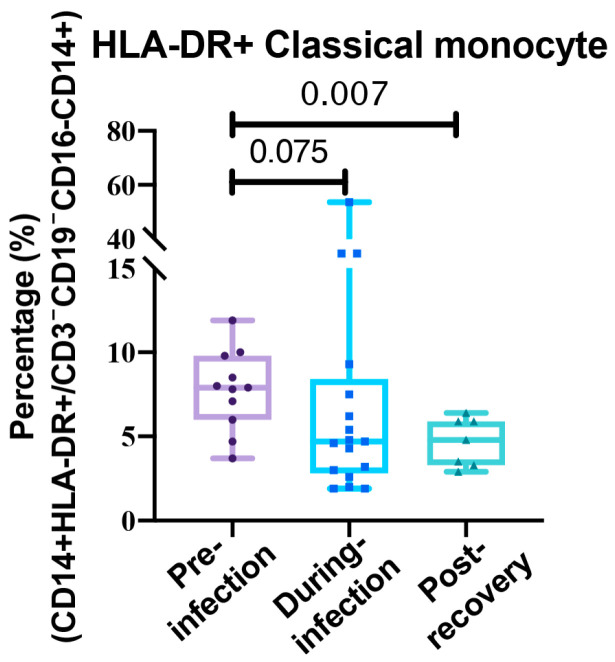
Box plots depict the distribution of HLA-DR+ classical monocyte proportions among total monocyte in lung cancer patients before (*n* = 11), during (*n* = 17), and after recovery from (*n* = 7) SARS-CoV-2 infection. Comparisons were performed using Student’s *t*-test.

We first compared the data collected during the pre-infection and during-infection periods. Following SARS-CoV-2 infection, all 14 cytokines we measured exhibited varying degrees of elevation. These included IL-1β (*p* < 0.0001), IL-2 (*p* = 0.0078), IL-4 (*p* = 0.0008), IL-5 (*p* < 0.0001), IL-6 (*p* = 0.0012), IL-8 (*p* = 0.0048), IL-10 (*p* < 0.0001), IL-12p70 (*p* = 0.0002), IL-17A (*p* = 0.0048), IL-17F (*p* = 0.0301), IL-22 (*p* = 0.0003), TNF-α (*p* = 0.002), TNF-β (*p* = 0.0041), and IFN-γ (*p* = 0.0075). This indicated that patients experienced a systemic cytokine storm during the acute phase of infection (Figure 2). In the analysis of whole-blood immune-cell subsets, most proportions remained statistically unchanged during acute infection (Supplementary Figure S2). One exception was HLA-DR+ monocytes: their peripheral-blood proportion dropped markedly after infection compared with pre-infection levels, although this decrease did not reach statistical significance (*p* > 0.05) (Figure 3).

### 3.3. The Lingering Echoes of Infection

**Figure 4:**
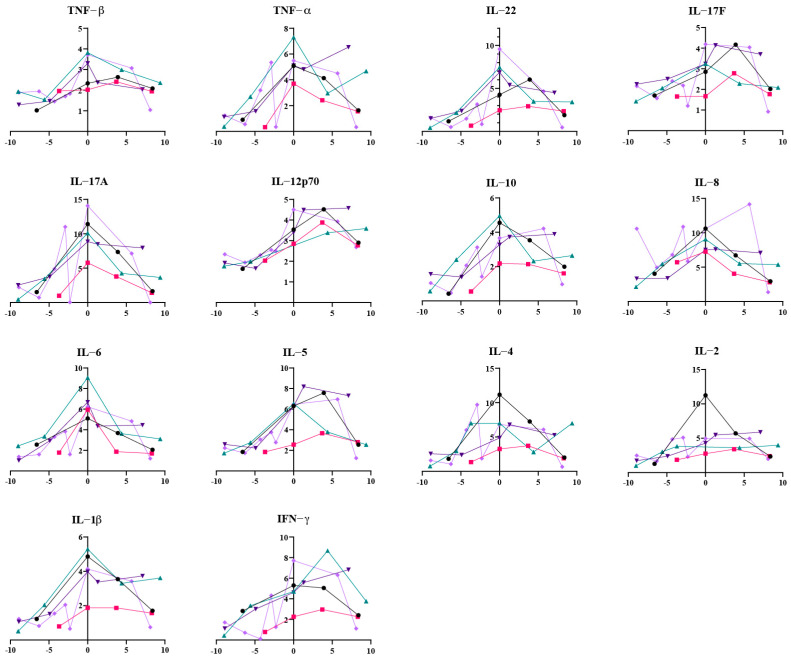
Plasma inflammatory cytokine trajectories in lung cancer patients with SARS-CoV-2 infection (*n* = 5). *Y*-axis: plasma cytokine concentration (pg/mL). *X*-axis: time relative to SARS-CoV-2 diagnosis; negative values indicate pre-diagnosis; positive values indicate post-diagnosis (range < ±10 weeks). Each colored line represents one patient; patient-specific colors are maintained across all panels.

**Figure 5:**
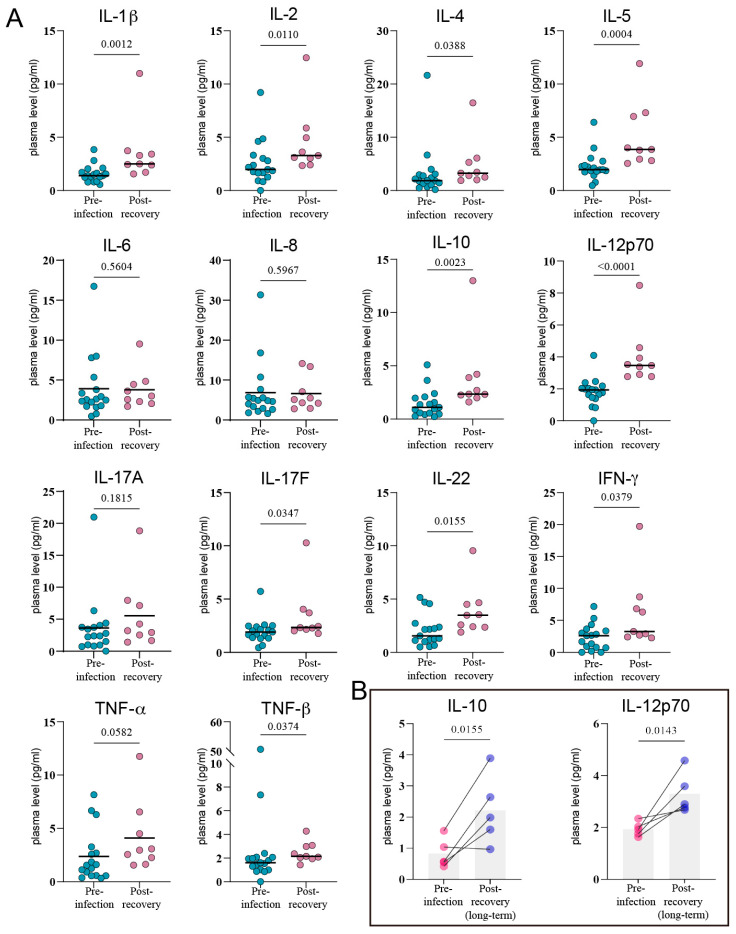
Comparison of plasma cytokine levels in lung cancer patients before SARS-CoV-2 infection versus after recovery. (**A**) Levels of 14 cytokines before infection (Before; *n* = 17; green) and at the first post-recovery measurement (After; *n* = 9; purple). Black bars indicate mean values (**B**) Plasma IL-10 and IL-12p70 levels before infection and approximately 10 weeks after recovery. Statistical analysis was performed using paired *t* test.

Perhaps more striking was the finding that this immune disruption did not simply end with viral clearance. We plotted the longitudinal plasma cytokine measurements for 5 patients with complete data from pre-infection, during-infection, and post-recovery periods into line graphs (Figure 4). It was observed that the levels of most cytokines decreased after patients recovered. However, we found that these cytokine levels did not return to their pre-infection baseline. A comparison between cytokine levels during infection and the first measurement post-recovery revealed that plasma levels of IL-1β (*p* = 0.0012), IL-2 (*p* = 0.0110), IL-4 (*p* = 0.0388), IL-5 (*p* = 0.0004), IL-10 (*p* = 0.0023), IL-12p70 (*p* < 0.0001), IL-17F (*p* = 0.0347), IL-22 (*p* = 0.0155), TNF-β (*p* = 0.0374), and IFN-γ (*p* = 0.0379) remained significantly elevated after recovery from SARS-CoV-2 infection (Figure 5A). With an extended observation period, it was evident that most of these cytokines continued to decline over time, eventually approaching baseline levels (Figure 4). Interestingly, the effects of SARS-CoV-2 infection on plasma IL-10 and IL-12p70 appeared to be particularly prolonged. At nearly 10 weeks post-recovery, plasma IL-10 and IL-12p70 levels remained significantly higher than their baseline values (IL-10, *p* = 0.0155; IL-12p70, *p* = 0.0143) (Figure 5B). The dynamic curves for some patients even suggested that their plasma IL-10 and IL-12p70 levels showed no downward trend (Figure 4).

The analysis of immune cell subsets showed that the proportions of cell populations that were not significantly altered during the acute infection phase also remained stable post-recovery (Supplementary Figure S2). Consistent with the prolonged immune dysregulation, the proportion of HLA-DR+ monocytes in whole blood did not recover to baseline levels even after recovery (7.76% [SD: 5.63] vs. 4.67% [SD: 2.07], *p* = 0.0071) (Figure 3).

### 3.4. The Clinical Verdict: Does COVID-19 Alter the Efficacy of Immunotherapy?

**Figure 6:**
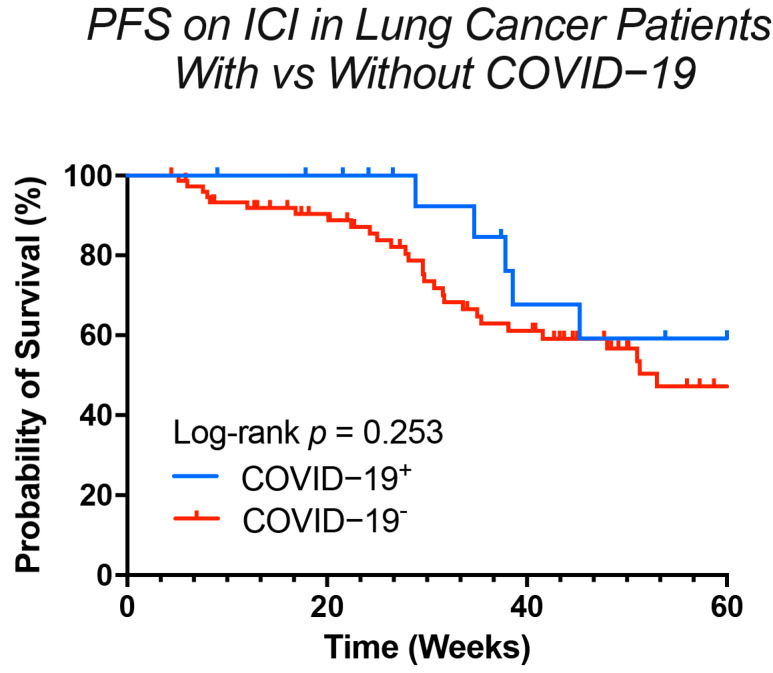
Progression-free survival on immune-checkpoint inhibitors in lung cancer patients with versus without COVID-19. Survival curves were compared by the log-rank test.

Immune Checkpoint Inhibitors (ICIs) are a widely used treatment for lung cancer that enhance the body’s anti-tumor immune response by activating the immune system. Given that COVID-19 infection can alter the patient’s immune environment, we investigated its potential impact on the efficacy of ICI therapy. To do so, we identified a subgroup of patients receiving ICI-based regimens, which included ICI monotherapy or ICIs in combination with chemotherapy or targeted therapy. From a pool of lung cancer patients treated during the same period but without a history of COVID-19, we selected a control group of 76 patients. This control group was matched 1:4 to the COVID-19 cohort based on demographic and clinical characteristics, including age, sex, tumor histology, and TNM stage (Supplementary Table S3). We then conducted a survival analysis on both groups. The analysis measured Progression-Free Survival (PFS), defined as the time from the initiation of ICI therapy to disease progression or death from any cause, with an observation period extending beyond one year to April 2024. The PFS curves indicated that COVID-19 infection did not have a significant long-term impact on the efficacy of immunotherapy, as the survival curves for the two groups converged after 43 weeks (Figure 6). However, a notable separation of the curves was observed between 20 and 40 weeks, where the COVID-19 cohort appeared to experience a later onset of disease progression compared to the uninfected cohort. This result suggested that COVID-19 infection might have a short-term, and potentially positive, influence on the therapeutic effects of immunotherapy.

### 3.5. Hypothesizing the “Why”: Unlocking Predictive Clues

**Figure 7:**
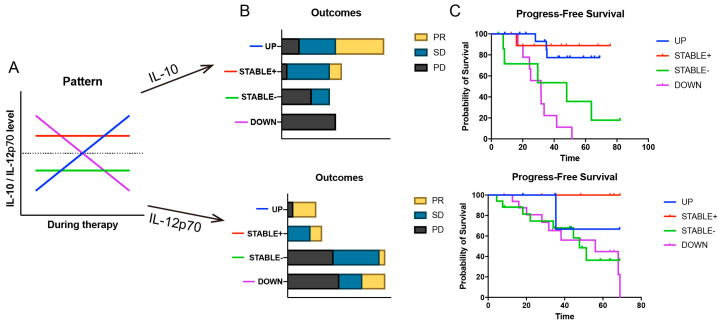
Association between plasma cytokine fluctuation patterns of IL-10 and IL-12p70 and ICI therapeutic outcomes. (**A**) Schematic illustration of the cytokine fluctuation patterns. (**B**) Distribution of ICI treatment responses across the different patterns. (**C**) Kaplan–Meier curves of progression-free survival for each pattern.

Numerous studies have reported associations between cytokine levels at baseline or during treatment and therapeutic response [26,27,28]. Our preceding findings suggested that the prolonged elevation of IL-10 and IL-12p70 induced by COVID-19 might exert a long-term influence on the efficacy of ICI therapy. To explore this hypothesis, we retrospectively analyzed the cytokine data of 76 non-infected lung cancer patients from the same period. We selected 29 patients who had at least three cytokine measurements recorded from baseline until disease progression. Upon observing the dynamic fluctuations of IL-10 and IL-12p70 during their treatment, we identified four distinct patterns: 1. UP: Plasma cytokine levels consistently increased after ICI initiation or experienced a peak exceedingly twice the baseline level. 2. STABLE+: Patients exhibited high baseline cytokine levels (>1.5 pg/mL) that were maintained with minor fluctuations throughout the treatment period. 3. STABLE-: Patients had low baseline cytokine levels (<1 pg/mL) that remained consistently low (<1.5 pg/mL) with minor fluctuations. 4. DOWN: Regardless of baseline levels, cytokine concentrations consistently decreased post-treatment, with multiple readings falling below 0.75 pg/mL. (Figure 7A) We stratified the patients into four groups based on these patterns. An analysis of their best treatment outcomes revealed that patients in the UP and STABLE+ pattern groups had a higher proportion of Partial Response (PR), whereas those in the DOWN and STABLE− pattern groups had a higher incidence of Progressive Disease (PD). (Figure 7B) Furthermore, a PFS analysis of these four groups demonstrated that patients with UP and STABLE+ patterns had longer PFS, while those with DOWN and STABLE- patterns had shorter PFS. When these patterns were consolidated into two larger groups (UP and STABLE+ vs. DOWN and STABLE-), the difference in PFS was statistically significant (*p* < 0.05) (Figure 7C).

## 4. Discussion

The advent of immune checkpoint inhibitors (ICIs) has revolutionized the treatment landscape for non-small cell lung cancer (NSCLC), yet the factors governing therapeutic response remain incompletely understood [29]. The interaction between viral infections and cancer immunotherapy represents a critical area of investigation, particularly in the wake of the COVID-19 pandemic, which introduced an unprecedented variable: a potent viral pathogen capable of profoundly modulating the host immune system [30,31]. Our study sought to elucidate how SARS-CoV-2 infection modulates the immune environment in lung cancer patients and its consequent impact on the efficacy of ICI therapy. Our investigation revealed a complex sequence of immunological events: an initial, acute cytokine storm characterized by the elevation of 14 distinct inflammatory mediators, followed by a prolonged period of immune disturbance. This complex immune modulation was associated with a paradoxical clinical outcome, where long-term progression-free survival (PFS) was not impaired, and a transient, statistically significant improvement in short-term disease control was observed.

The acute cytokine storm observed during SARS-CoV-2 infection in our cohort mirrors the inflammatory cascade documented in previous studies [32,33]. The elevation of multiple pro-inflammatory cytokines reflects the robust innate immune activation characteristic of the infection. This cytokine profile bears striking similarities to the inflammatory milieu observed in immune-related adverse events (irAEs) associated with ICI therapy, raising important questions about the potential synergistic or antagonistic effects of concurrent viral infection and immunotherapy [34,35]. Perhaps more striking than the acute storm was the persistence of cytokine elevation for weeks to months following acute infection, a novel finding with significant clinical implications. Previous studies have documented prolonged immune dysfunction following COVID-19, but the specific duration and pattern of cytokine elevation in cancer patients receiving immunotherapy had not been systematically characterized [36]. Our observation that specific cytokines, particularly IL-10 and IL-12p70, remained elevated for up to 10 weeks post-infection suggests that the immune perturbation induced by SARS-CoV-2 extends far beyond the acute phase of illness.

The relationship between COVID-19 and ICI efficacy presents a paradoxical picture that reflects the dual nature of immune activation [31,37]. Our survival analysis revealed no long-term detriment to PFS, which is consistent with several large retrospective studies [38,39]. However, we observed a statistically significant short-term improvement in disease control, suggesting that the immune activation induced by SARS-CoV-2 may, under certain circumstances, enhance anti-tumor responses. These findings position SARS-CoV-2 infection as a potential “double-edged sword.” While the profound immune activation could potentiate anti-tumor immunity, it also carries a theoretical risk of exacerbating irAEs [40,41,42]. It is noteworthy, however, that in our cohort, no patients discontinued ICI treatment due to severe irAEs. This fortunate circumstance allowed for an extended follow-up period and a more direct assessment of the impact of COVID-19 on ICI efficacy, unconfounded by treatment interruptions due to toxicity.

We hypothesize that the sustained elevation of IL-10 and IL-12p70 may provide a mechanistic basis for the observed transient clinical benefit. IL-12p70 is a canonical Th1-polarizing cytokine, and its role in promoting robust anti-tumor immunity is well-established [43]. It is critical for cytotoxic T-lymphocyte (CTL) activation, and its higher levels are often associated with improved ICI response [44]. IL-12 primarily acts by eliciting robust IFN-γ secretion from NK and T cells; in multiple pre-clinical tumor models, peak IFN-γ levels in both peripheral blood and the tumor bed are detectable within 6–24 h of IL-12 administration. IFN-γ is the most potent and best-characterized inducer of PD-L1, and its IL-12-driven release markedly up-regulates PD-L1 expression on tumor cells, macrophages and dendritic cells, thereby potentiating the efficacy of subsequent PD-1/PD-L1 blockade [45,46] (PMID: 32817076; PMID: 29034543). Concurrently, IFN-γ transcriptionally activates pro-inflammatory genes such as MHC-II and the chemokines CXCL9/10, which enhances T-cell trafficking and intratumoral infiltration [47] (PMID: 39034318).

The role of IL-10 is more nuanced. Traditionally viewed as an immunosuppressive factor, recent evidence suggests it can have context-dependent anti-tumor functions, particularly when administered in long-acting forms that can activate tumor-resident CD8+ T cells and reverse T cell exhaustion [43,48,49,50]. Animal experiments have shown that IL-10 promotes intra-tumoral CD8^+^ T cell expansion and enhances IFN-γ production, extending survival [51]. However, this therapeutic avenue requires further exploration, as a Phase II clinical trial of PEGylated IL-10 (pegilodecakin) combined with ICIs in metastatic NSCLC (NCT03382899/NCT03382912) failed to meet its primary endpoint, highlighting the complexity of its clinical application [52]. Our analysis of cytokine dynamics in a non-COVID cohort lends support to our hypothesis, as patients on ICI therapy whose IL-10 and IL-12p70 levels followed an “UP” or “STABLE+” trajectory exhibited significantly longer PFS. This suggests that SARS-CoV-2 infection may act as an unscheduled immune adjuvant, pushing a subset of patients into a favorable cytokine profile predictive of a better response to ICIs.

An additional intriguing finding was the significant and sustained reduction in peripheral monocyte proportions following COVID-19. As precursors to tumor-associated macrophages and dendritic cells, monocytes are pivotal in shaping the tumor microenvironment and orchestrating adaptive immunity [53]. Although the mechanism for this reduction is unclear, it may be clinically relevant. High monocyte counts have been associated with poorer outcomes in patients receiving ICIs across multiple tumor types [54]. Therefore, the observed reduction following COVID-19 may represent another mechanism contributing to the transient therapeutic benefit seen in our cohort. This observation warrants further investigation to understand its mechanistic drivers and clinical relevance.

We acknowledge several limitations to our study, including its retrospective design, the relatively small sample size, and the inherent heterogeneity of the patient population. These factors necessitate caution in the interpretation of our findings and underscore the need for validation in larger, prospective, multi-center cohorts.

In conclusion, our study provides a degree of reassurance regarding the continued use of ICIs in lung cancer patients with a history of SARS-CoV-2 infection, demonstrating no compromise to long-term survival. More importantly, these findings carry broader clinical implications. They underscore the importance of considering the impact of other infectious diseases on patients undergoing immunotherapy, as these events may act as potent, unscheduled immune modulators [55]. Furthermore, our work highlights the potential of monitoring dynamic changes in cytokines, such as IL-10 and IL-12p70, as a prognostic tool to predict patient outcomes and guide clinical management. Looking forward, this research opens new avenues for exploring whether the controlled modulation of specific cytokine pathways could serve as an adjuvant strategy to augment the efficacy of cancer immunotherapy, ultimately personalizing treatment and improving patient outcomes.

## Data Availability

All datasets are available from the corresponding author for reasonable request.

## Supplementary Materials

The following supporting information can be downloaded at: https://www.mdpi.com/article/10.3390/v17101314/s1, Figure S1: Gating strategy for whole-blood immunophenotyping by flow cytometry; Figure S2: Longitudinal comparison of peripheral-blood lymphocyte-subset percentages before, during and after SARS-CoV-2 infection; Table S1: Demographic of COVID-19 cohort for detection of immune cell subsets: title; Table S2: Demographic of COVID-19 cohort for detection of inflammatory cytokines; Table S3: Table of variables in terms of age, sex, histological classification, TNM in two groups (lung cancer with COVID-19 and lung cancer).

## Abbreviations

The following abbreviations are used in this manuscript:

irAEs	Immune-related adverse events
CTL	Cytotoxic T-lymphocyte
ICI	Immune checkpoint inhibitor
PFS	Progression-free survival
NSCLC	Non-small cell lung cancer
TME	tumor microenvironment
CRS	cytokine release syndrome
