# Supplementary material for: SARS-CoV-2 Infection Alters the Immune Microenvironment in Lung Cancer Patients Undergoing Immunotherapy and Affects Treatment Outcomes
Source: Viruses. 2025 Sep 28;17(10):1314. doi: 10.3390/v17101314 (PMC12568021; doi:10.3390/v17101314)
Supplement: Supplementary file 1 [file viruses-17-01314-s001.zip › viruses-3877098-supplementary.pdf]

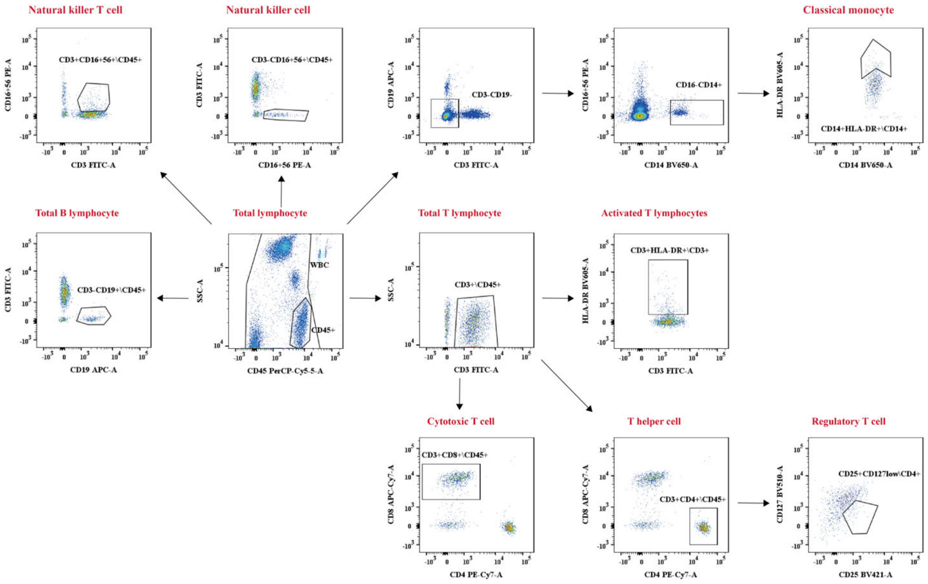

Figure S1. The gating strategy of the whole blood immunophenotyping assay.

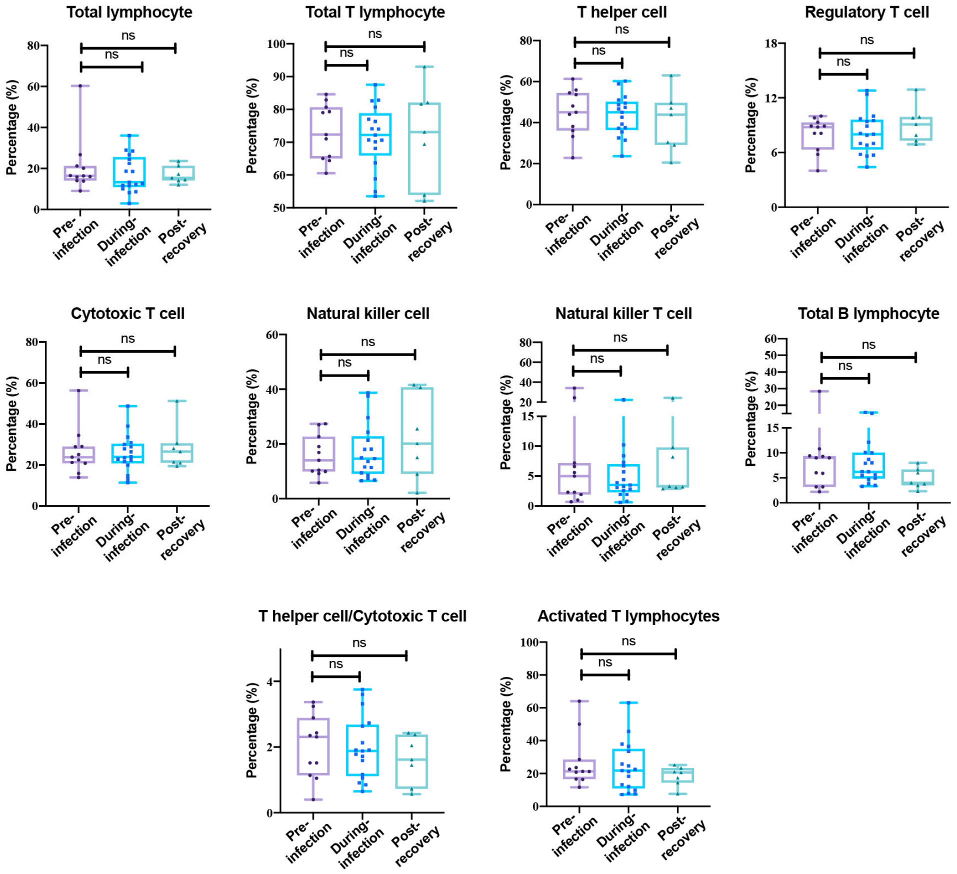

Figure S2. Distribution of peripheral blood immune-cell subset proportions in lung cancer patients before, during, and after SARS-CoV-2 infection. Comparisons were performed with Student's t-test; ns, not significant.

Table S1. Demographic of COVID-19 cohort for detection of immune cell subsets.

Patient No.	Age	Sex	Pathological types	Staging	TNM	Time of diagnosis	Detection time of immune cell subsets			Therapies	Time to start treatment	Observation outcome	Time of disease progression or
							Preinfection	At infection	Postinfection				

			of lung cancer			COVID-19							end of observation
**1**	**64**	**M**	**LUAD**		**T3N3M1**	**2022/12/2**	**2022/11/11**	**2022/12/2**	**2022/12/30**	CI	2022/9/1	SD	2024/1/22
2	62	M	LUSC	IIIa	T2N2M0	2022/12/4	2022/11/11	2022/12/4	-	N	2022/11/9	SD	2023/11/25
3	44	M	LUSC	Ib	T2N0M0	2022/12/9	2022/11/1	2022/12/9	2023/2/24	CI	2022/10/31	PD	2023/7/28
4	65	M	LUAD	IVb	T2N3M1	2022/12/13	2022/10/25	2022/12/13	2023/1/31	CI	2022/3/24	SD	2023/7/28
5	60	M	LUAD	IIb	T2N1M0	2022/12/13	-	2022/12/13	-	C	2022/12/5	PD	2023/8/11
6	65	M	LUSC		T3N0M0	2022/12/14	-	2022/12/14	-	CI	2022/12/16	SD	2023/2/17
7	69	M	CA	IV	T4N2M1	2022/12/19	-	2022/12/19	-	T	2021/9/29	PD	2022/10/24
8	55	M	LUSC	III	T4N3M0	2022/12/23	2022/11/4	2022/12/23	-	CI	2022/3/21	PD	2023/2/1
9	56	M	LUSC	IVa	T4N2M1b	2022/12/26	-	2022/12/26	-	CI	2022/9/30	SD	2023/2/2
10	67	M	SCLC	IV	T4N2M1	2022/12/27	2022/11/22	2022/12/27	-	C	2022/10/31	PD	2023/4/7
11	68	M	LUSC	IIb	T2N1M0	2022/12/28	2022/10/25	2022/12/28	2023/2/24	CI	2022/9/26	SD	2023/3/31
12	72	M	LUAD	IV	T4N3M1	2022/12/28	2022/11/11	2022/12/28	-	CI	2022/1/12	SD	2024/2/19
13	68	M	SCLC	IV	T4N3M1	2022/12/28	2022/11/4	2022/12/28	-	CE	2022/11/10	PD	2023/1/18
14	67	M	SCLC	IV	T2N3M1	2022/12/29	-	2022/12/29	2023/1/31	CI	2022/10/19	PD	2023/6/19
15	67	M	SCLC			2023/1/6	2022/11/8	2023/1/6	2023/2/24	CI	2022/6/23	PR	2024/2/2
16	69	F	SCLC			2022/12/12	2022/11/4	2022/12/12	2023/1/10	CI	2022/9/20	PR	2023/3/8
17	63	M	LUAD			2023/5/17	-	2023/5/12	-	N	2023/6/5	SD	2023/7/15

F, female; M, male; SCLC, small cell lung cancer; LUAD, lung adenocarcinoma; LUSC, lung squamous cell carcinoma; CA, lung cancer; PR, partial response; PD, progressive disease; SD, stable disease; CI, chemotherapy combined with immunotherapy; C, chemotherapy; I: immunotherapy; T, targeted therapy; CIT, chemotherapy combined with immunotherapy and targeted therapy; N, neoadjuvant treatment.

Table S2. Demographic of COVID-19 cohort for detection of inflammatory cytokines.

**Patient**	**Age**	**Sex**	**Pathological types of lung cancer**	**Staging**	**TNM**	**Time of diagnosis of COVID-19**	**Detection time of inflammatory cytokines**			Therapies	Time to start treatment	Observation outcome	Time of disease progression or end of observation
							**Preinfection**	**At infection**	**Postinfection**				

**2**	**62**	**M**	**LUSC**	**IIIa**	**T2N2M0**	**2022/12/4**	**2022/11/10**	**2022/12/4**	**-**	N	2022/11/9	SD	2023/11/25
3	64	M	LUAD		T3N3M1	2022/12/2	2022/10/17	2022/12/2	2023/1/30	CI	2022/9/1	SD	2024/1/22
3	44	M	LUSC	Ib	T2N0M0	2022/12/9	2022/11/3	2022/12/9	2023/2/23	CI	2022/10/31	PD	2023/7/28
4	65	M	LUAD	IVb	T2N3M1	2022/12/13	2022/10/27	2022/12/13	2023/2/2	CI	2022/3/24	SD	2023/7/28
5	60	M	LUAD	IIb	T2N1M0	2022/12/13	-	2022/12/13	-	C	2022/12/5	PD	2023/8/11
6	65	M	LUSC		T3N0M0	2022/12/14	-	2022/12/14	-	CI	2022/12/16	SD	2023/2/17
7	69	M	CA	IV	T4N2M1	2022/12/19	-	2022/12/19	-	T	2021/9/29	PD	2022/10/24
8	55	M	LUSC	III	T4N3M0	2022/12/23	2022/11/3	2022/12/23	-	CI	2022/3/21	PD	2023/2/1
10	67	M	SCLC	IV	T4N2M1	2022/12/27	2022/11/21	2022/12/27	-	C	2022/10/31	PD	2023/4/7
11	68	M	LUSC	IIb	T2N1M0	2022/12/28	2022/10/24	2022/12/28	2023/2/23	CI	2022/9/26	SD	2023/3/31
12	72	M	LUAD	IV	T4N3M1	2022/12/28	2022/11/14	2022/12/28	-	CI	2022/1/12	SD	2024/2/19
13	68	M	SCLC	IV	T4N3M1	2022/12/28	2022/11/3	2022/12/28	-	C	2022/11/10	PD	2023/1/18
14	67	M	SCLC	IV	T2N3M1	2022/12/29	-	2022/12/29	2023/1/30	CI	2022/10/19	PD	2023/6/19
16	69	F	SCLC			2022/12/12	2022/11/3	2022/12/12	2023/1/12	CI	2022/9/20	PR	2023/3/8
17	63	M	LUAD			2023/5/17	-	2023/5/15	-	N	2023/6/5	SD	2023/7/15
18	53	M	LUSC	IIIa	T2N2M0	2022/12/4	2022/10/20	2022/12/4	-	N	2022/9/7	SD	2023/3/27
19	72	F	LUAD		T3N0M0	2022/12/21	2022/11/17	2022/12/21	2023/2/9	CIT	2021/7/8	SD	2023/9/27
20	66	M	LUSC	IV	T4N3M1	2022/12/21	2022/11/7	2022/12/21	2023/1/30	CI	2022/9/29	PD	2023/6/21
21	59	M	SCLC	IVa	T2N2M1	2022/12/3	2022/11/7	2022/12/3	2023/1/30	CI	2022/10/14	PR	2023/7/3
22	50	M	LUSC			2023/1/5	-	2023/1/5	-	CI	2022/10/25	PD	2023/5/15
23	68	M	LUSC	III	T3N3M0	2023/1/9	-	2023/1/9	-	C	2022/9/28	PD	2023/6/29
24	70	M	LUSC	II	T2N0M0	2023/1/9	-	2023/1/9	-	N	2020/10/16	SD	2021/11/18
25	68	F	LUSC		T3N3M1	2023/1/11	-	2023/1/11	-	-	-	-	-
27	41	F	LUAD	IVb	T1cN0M1c	2023/5/25	2023/3/30	-	-	CI	2023/4/13	PR	2023/10/25
28	68	M	LUSC		T4N0M1a	2023/5/29	2023/4/13	2023/5/29	-	CI	2023/2/10	PR	2024/2/2

29	67	F	LUAD	T2N1Mx	2023/5/22	2023/5/11	-	-	T	2023/5/30	PR	2023/11/2
30	71	F	LUAD IVa	T4N0M1a	2023/5/22	-	2023/5/18	-	T	2023/5/15	SD	2023/7/17
31	73	F	LUSC	T4N2M1c	2023/5/22	-	2023/5/22	-	CI	2023/4/24	SD	2023/9/22

F, female; M, male; SCLC, small cell lung cancer; LUAD, lung adenocarcinoma; LUSC, lung squamous cell carcinoma; PR, partial response; PD, progressive disease; SD, stable disease; CI, chemotherapy combined with immunotherapy; C, chemotherapy; I: immunotherapy; T, targeted therapy; CIT, chemotherapy combined with immunotherapy and targeted therapy; N, neoadjuvant treatment.

Table S3. Table of variables in terms of age, sex, histological classification, TNM in two groups (lung cancer with COVID-19 and lung cancer)

Variables	Lung cancer with COVID-19	Lung cancer
Total	18	76
Age, y	64.28±8.48	64.07±7.46
Sex, male, n (%)	14(77.8%)	62(81.6%)
LUAD, n (%)	4(22.2%)	27(35.5%)
LUSC, n (%)	9(50.0%)	28(36.8%)
SCLC, n (%)	4(22.2%)	19(25.0%)
Others[1], n (%)	1(5.6%)	2(2.6%)
II stage, n (%)	2(11.1%)	4(5.3%)
III stage, n (%)	1(5.6%)	13(17.1%)
IV stage, n (%)	11(61.1%)	49(64.5%)
Others[2], n (%)	4(22.2%)	10(13.2%)

1：histological classification was unknown；2：TNM stage is unknown. LUAD: lung adenocarcinoma; LUSC: lung squamous carcinoma; SCLC: small cell lung cancer.
